# Crack Monitoring in Rotating Shaft Using Rotational Speed Sensor-Based Torsional Stiffness Estimation with Adaptive Extended Kalman Filters

**DOI:** 10.3390/s23052437

**Published:** 2023-02-22

**Authors:** Young-Hun Park, Hee-Beom Lee, Gi-Woo Kim

**Affiliations:** Department of Mechanical Engineering, Inha University, Incheon 22212, Republic of Korea

**Keywords:** crack monitoring, rotating shaft, torsional stiffness estimation, rotational speed sensors, adaptive extended Kalman filter, forgetting factor update

## Abstract

In this study, we present an alternative solution for detecting crack damages in rotating shafts under torque fluctuation by directly estimating the reduction in torsional shaft stiffness using the adaptive extended Kalman filter (AEKF) algorithm. A dynamic system model of a rotating shaft for designing AEKF was derived and implemented. An AEKF with a forgetting factor (*λ*) update was then designed to effectively estimate the time-varying parameter (torsional shaft stiffness) owing to cracks. Both simulation and experimental results demonstrated that the proposed estimation method could not only estimate the decrease in stiffness caused by a crack, but also quantitatively evaluate the fatigue crack growth by directly estimating the shaft torsional stiffness. Another advantage of the proposed approach is that it uses only two cost-effective rotational speed sensors and can be readily implemented in structural health monitoring systems of rotating machinery.

## 1. Introduction

Rotating machinery (or turbomachinery) has steadily been in the field of interest for industrial applications in internal combustion engines, power generators, turbines, and high-speed machining [1]. Rotating machinery generally consists of a rotor and a non-rotating part (stator), with torque transmitted through a rotating shaft. Cracks in rotary shafts are among the most dangerous and significant defects. The crack occurs in rotating shafts because of various mechanisms such as high and low cycle fatigue, stress corrosion, or unbalanced force caused by the rotor offset [2]. The shafts of the above-mentioned machines are typically subjected to harsh working conditions, such as loading and temperature variations. Thus, successive failures can lead to enormous economic and human resource losses. If a crack propagates continuously and is not detected in advance, an abrupt failure may occur, leading to catastrophic consequences. Thus, real-time monitoring of crack damage in the rotating shaft is essential.

Generally, contact sensors, which provide high data accuracy and convenience, can be used to detect such cracks in a rotating shaft. However, rotating and internal parts are generally difficult to measure directly. Thus, it is challenging to monitor shaft cracks using a contact sensor, such as a strain gauge. Therefore, in recent years, fault diagnosis studies on rotating machinery have focused on indirect detection methods through vibration response characteristic analysis of components, such as bearings and gears. As a result, numerous vibration-based crack detection techniques have been developed over the last decades [3]. These techniques include experimental signal-based and model-based methods. Several model-based crack detection methods, such as wavelet transform [4], have been developed to enhance fault diagnosis. Experimental signal-based methods using nonlinear vibration responses have also been widely used for damage detection in structures [5,6]. 

For more precise, reliable, and effective detection, various non-destructive techniques (NDT), such as radiography, magnetic particle inspection, and ultrasonic methods, are attempted to diagnose and monitor the behavior of rotating machines, although these techniques consume more time and are expensive [7]. However, high-frequency amplitudes are too small to detect cracks, and responses can be generated by assembly tolerances, manufacturing state noise, and other defects.

These disadvantages of the current technology necessitate developing non-traditional technology for detecting structural surface damage, such as cracks in the rotating shaft [8,9,10,11,12]. Non-model-based crack detection has also been attempted as a statistical-based data analysis method, using trained models from artificial neural networks [13] and genetic algorithms [14]. Recently, machine learning has been studied as a solution for detecting defects effectively without human experts [15]. However, this method is data-inefficient because we cannot acquire sufficient experimental data on actual crack sequences for large systems. Over the last decades, some research on structural health monitoring has been conducted based on the adaptive extended Kalman filter algorithm (AEKF) [16,17,18,19,20]. For example, the Kalman filter with the forgetting factor method had been applied to several systems, such as a lithium-ion battery, to consider the variation of system model parameters [21]. However, it is still necessary to study a new detection method, although previous studies have shown promising results in detecting cracks in rotating shafts.

**Figure 1:**
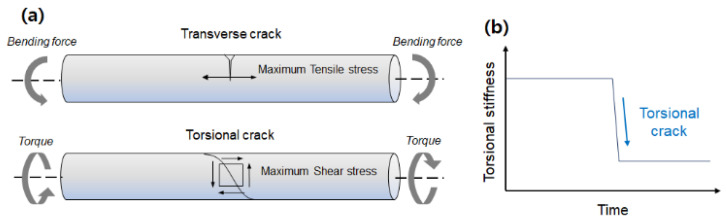
Characterization of fatigue cracks: (**a**) two types of crack propagation: transverse and torsion crack, (**b**) sudden torsional stiffness reduction.

Therefore, this study primarily aims to provide an alternative solution for detecting crack damages in rotating shafts by directly estimating the change in stiffness using the adaptive extended Kalman filter algorithm (AEKF) with a forgetting factor update. To the best of our knowledge, we report for the first time that it is possible to achieve a new means of detecting the torsional crack in a rotating shaft using AEKF with a forgetting factor update algorithm. Cracks of varying geometry are caused by different types of stress-field directions and are classified according to their orientation with respect to the shaft axis, as shown in Figure 1a. The direction of the stress field depends on the type of stress (such as bending or torsion) and geometric factors. When high cyclic stress is repeated, the crack propagates such that the crack plane is perpendicular to the direction of the tensile stress field. When bending stress is applied to the shaft, a stress field forms along the axis, and the crack propagates into the shaft section, creating a transverse crack, which is frequently called a breathing crack [22]. Torsional stress forms a tensile stress field in the direction of 45° to the shaft axis. In this study, we focused on torsional slant cracks of shafts and attempted to use Kalman-filter-based torsional stiffness estimation. When fatigue cracks occur in rotating shaft systems under alternating torque excitation, the cracks gradually grow larger over time as they are repeatedly opened and closed. As the cross-sectional area decreases, the torsional stiffness of the shaft suddenly decreases, as shown in Figure 1b. The AEKF-based estimator of shaft torsional stiffness using a dynamic model of rotating machinery is described in Section 2. Simulation results using the proposed algorithm under sinusoidal torque input are presented in Section 3. The simulation results were experimentally validated, as described in Section 4.

## 2. Design of Adaptive Extended Kalman Filters

### 2.1. Dynamic Modeling of Rotating Shaft

**Figure 2:**
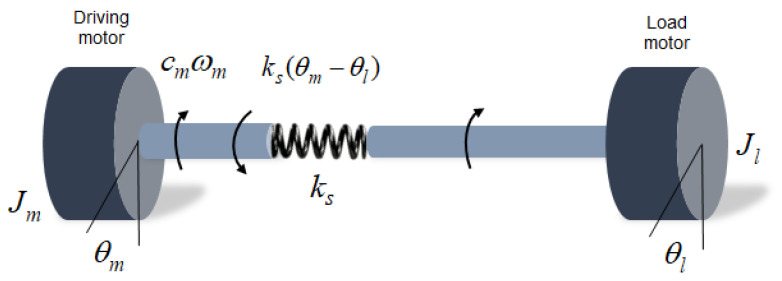
Schematic of the rotating shaft model with shaft torsional stiffness.

Although there are various connecting structures, such as bearings and shafts, in a real rotating shaft system, the system model was formulated based on a dynamic circular shaft, to which torque and rotational speed were applied together. It is assumed to be a lumped-parameter model with two primary masses (i.e., a semi-definite system with a single natural frequency) because it is unlikely to excite the higher modes in our simple test bed system (single frequency excitation). The system elements simulated the driving-load motor dynamo for experimental verification of the proposed algorithm. The stiffness of the bellows coupling connecting the shaft and the damping effect of the bearing stand were neglected. As shown in Figure 2, the rotating shaft model comprised four components. The governing equation for the shaft rotation is given as follows:

(1) $$J_{m}{\ddot{\theta }}_{m}+c_{m}{\dot{\theta }}_{m}+k_{s}(\theta_{m}-\theta_{l})=T_{m},$$

(2) $$k_{s}(\theta_{m}-\theta_{l})-J_{l}{\ddot{\theta }}_{l}=0,$$

where $J_{m}$ is the moment of inertia (driving motor), $J_{l}$ is the moment of inertia (load motor), $k_{s}$ is the torsional stiffness of the shaft, and $c_{m}$ is the damping coefficient of the viscous friction of the driving motor. When torque is applied by the load motor, the angular velocity difference between the two sides is caused by the stiffness of the shaft connecting the two motors. It is necessary to express the dynamic model into a state-space model to implement the Kalman filter algorithm.

(3) $$\dot{x}=Ax+Bu,$$

(4) y=Cx+Du,

where *x* is the state vector, *u* is the input vector, and $\dot{x}$ is the time derivative of the state vector. In Equation (3), *A* is a state matrix, and *B* is an input matrix. In Equation (4), *y* is a measurement variable, *C* is a measurement matrix, and *D* is a feed-forward matrix.

The four state variables for stiffness estimation are selected as shown in Equation (5). In this study, the time-varying stiffness is treated as a state variable, and it is assumed to be linearly proportional to the crack sizes for designing Kalman filters, although it can be changed by the nonlinear dynamics of the rotating shaft system [23]. $\theta_{m}-\theta_{l}$ is the difference in angular displacement on both sides and $\omega_{l}$ is the angular velocity of the load motor. The fourth state variable is the angular velocity of the driving motor. The driving motor torque is an input for the system. From Equations (1) and (2), the state space equation was derived as follows:

(5) $$x={\left[x_{1}x_{2}x_{3}x_{4}\right]}^{T}={\left[\left(\theta_{m}-\theta_{l}\right)\omega_{l}k_{s}\omega_{m}\right]}^{T},$$

(6) $$u=T_{m},$$

(7) $$\dot{x}=f(x,u)=\left[\begin{array}{c} {\dot{x}}_{1}\\ {\dot{x}}_{2}\\ {\dot{x}}_{3}\\ {\dot{x}}_{4} \end{array}\right]=\left[\begin{array}{c} x_{4}-x_{2}\\ \frac{x_{3}x_{1}}{J_{l}}\\ 0\\ \frac{-c_{m}x_{4}-x_{3}x_{1}+u}{J_{m}} \end{array}\right],$$

(8) $$y=h(x)=\left[\begin{array}{c} x_{2}\\ x_{4} \end{array}\right]=\left[\begin{array}{cccc} \begin{array}{l} 0\\ 0 \end{array} & \begin{array}{l} 1\\ 0 \end{array} & \begin{array}{l} 0\\ 0 \end{array} & \begin{array}{l} 0\\ 1 \end{array} \end{array}\right]\left[\begin{array}{c} \theta_{m}-\theta_{l}\\ \omega_{l}\\ k_{s}\\ \omega_{m} \end{array}\right].$$

The reformulated system model f(x,u) is a nonlinear model, and the measurement model *h(x)* is a linear model with an actual measurable angular velocity value as the output. For the estimation of torsional stiffness, an extended Kalman filter (EKF) that linearizes a nonlinear model is required, and an adaptive EKF (AEKF) with a P-adaptive loop is proposed to improve estimation performance. As the proposed AEKF algorithm is based on the discrete-time domain, the continuous equation was discretized using the Euler method, as shown in Equation (9).

(9) $$\dot{x}=\frac{x(k)-x(k-1)}{\Delta t}\;\to \;x(k)=\dot{x}(k)\Delta t+x(k-1),$$

where Δ*t* is the time step, and *k* and *k* − 1 represent the time instant at *t = k*Δ*t* and *t* = (*k* − 1)Δ*t*, respectively. Substituting Equation (7) into Equation (9), Equations (10) and (11) are defined as follows:

(10) $$\left\{\begin{array}{l} x_{k}=f_{k-1}(x_{k-1},u_{k-1})\\ y_{k}=h_{k}(x_{k}) \end{array}\right..$$

### 2.2. Adaptive Extended Kalman Filters

Kalman filtering is a state-estimation technique developed by Rudolf Kalman in 1960. It features a recursive structure and optimally estimates the state of a linear dynamic system based on measurements contaminated by noises. Kalman filters are used in many industrial fields, such as computer vision, robotics, and vehicular electronics [24,25]. The general linear discrete-time system model required to design the KF is given as

(11) $$\begin{array}{l} x_{k+1}=Ax_{k}+Bu_{k}+w_{k}\\ y_{k}=Hx_{k}+v_{k} \end{array},$$

where $w_{k}$ is a multivariate Gaussian distribution system noise variable with a covariance matrix, and $v_{k}$ is a multivariate Gaussian distribution measurement noise variable with a covariance matrix. In this study, there was no input in the measurement model, and the application of the EKF was based on the nonlinear model. The general discrete-time equation is as follows:

(12) $$\left\{\begin{array}{l} x_{k}=f_{k-1}(x_{k-1},u_{k-1})+w_{k-1}\\ y_{k}=h_{k}(x_{k})+v_{k} \end{array}\right..$$

The extended Kalman filter assumes differentiability of the state-change function instead of linearity of the model. The nonlinear system model was linearized using the Jacobian, and the Jacobian matrix was calculated based on the previous estimate.

(13) $$A_{k-1}={\left.\frac{\partial f_{k-1}}{\partial x}\right|}_{{\hat{x}}_{k-1}}B_{k-1}={\left.\frac{\partial f_{k-1}}{\partial u}\right|}_{{\hat{x}}_{k-1}},$$

(14) $$H(k)={\left.\frac{\partial h_{k}}{\partial x}\right|}_{{\hat{x}}_{k|k-1}}.$$

Matrices A and B of the rotating shaft system model linearized using Equations (13) and (14) are as follows:

(15) $$A^{*}=\frac{\partial f(x,u)}{\partial x}=\left[\begin{array}{cccc} 0 & -1 & 0 & 1\\ \frac{x_{3}}{J_{L}} & 0 & \frac{x_{1}}{J_{L}} & 0\\ 0 & 0 & 0 & 0\\ -\frac{x_{3}}{J_{m}} & 0 & -\frac{x_{1}}{J_{m}} & \frac{C_{m}}{J_{m}} \end{array}\right],$$

(16) $$B^{*}=\frac{\partial f(x,u)}{\partial u}={\left[\begin{array}{cccc} 0 & 0 & 0 & \frac{1}{J_{m}} \end{array}\right]}^{T},$$

(17) $$H=\left[\begin{array}{l} \begin{array}{cccc} 0 & 1 & 0 & 0 \end{array}\\ \begin{array}{cccc} 0 & 0 & 0 & 1 \end{array} \end{array}\right],$$

where (*) indicates the system model matrix linearized using the Jacobian.

The discrete-time EKF algorithm has the following form:

▪ Initial estimation stage at k=0

(18) $$\left\{\begin{array}{l} {\hat{x}}_{0}=E[x_{0}]\\ P_{0}=E[(x_{0}-{\hat{x}}_{0}){\left(x_{0}-{\hat{x}}_{0}\right)}^{T}] \end{array}\right..$$

where *E* represents the expected value of the random variable.

▪ Prediction stage

(19) $$\begin{array}{l} \hat{x}(k|k-1)=f_{k-1}({\hat{x}}_{k-1},u_{k-1},0)\\ P(k|k-1)=A(k,k-1)P(k-1)A^{T}(k,k-1)+Q(k-1) \end{array}.$$

*A* matrix was differentiated using the Jacobian in Equation (13). In the prediction stage, the variables predicted are a priori state variable and an error covariance matrix.

▪ Correction stage

(20) $$K(k)=P(k|k-1)H^{T}(k){\left(H(k)P(k|k-1)H^{T}(k)+R(k)\right)}^{-1},$$

(21) $$\begin{array}{l} \hat{x}(k)=\hat{x}(k|k-1)+K(k)[y(k)-h_{k}({\hat{x}}_{k|k-1},u_{k},0)]\\ P(k)=[I-K(k)H(k)]P(k|k-1) \end{array}.$$

In general, it is difficult to estimate the time-varying parameter (shaft stiffness) using the EKF because filter estimation relies on past data, and state estimation can diverge when past data are not adequate for recursive estimation methods. In this study, an AEKF with a forgetting factor (*λ*) was used to resolve this technical limitation [26]. The updated forgetting factor corrects the error covariance matrix, and the Kalman gain matrix is increased by the inverse of the forgetting factor. In general, the forgetting factor is considered a constant tuning parameter. However, convergence decreases when the uncertainty is large, such as in a nonlinear model. In this study, an adaptive loop was employed for more weighting to recent data using the residual between the measured and estimated values [27,28]. The AEKF equation is identical to the EKF in Equation (19), except for the forgetting factor in the error covariance equation.

(22) $$P(k+1|k)=\lambda (k+1)A(k+1,k)P(k)A^{T}(k+1,k)+Q(k),$$

with λ(k)≥1. Thus, divergence is prevented by considering the influence of the most recently measured data on the state and parameter. The performance of the AEKF is the most important factor because it completely depends on the forgetting factor. The residual z(k) is defined as the difference between the measured and predicted values of the measurement. The residual is a white noise sequence when the optimal filtering gain is used.

(23) $$z(k)=y(k)-H(k)\hat{x}(k|k-1).$$

For any gain, the covariance of the residuals is expressed as:

(24) $$C_{0}(k)=E[z(k)z^{T}(k)]=H(k)P(k|k-1)H^{T}(k)+R(k).$$

The auto covariance of the residual is

(25) $$\begin{array}{l} C_{j}(k)=E[z(k+j)z^{T}(k)]\\ \;\;\;\;\;\;=H(k+j)A(k+j,k+j-1)\\ \;\;\;\;\times [I-K(k+j-1)H(k+j-1)]\cdots A(k+2,k+1)\\ \;\;\;\;\times [I-K(k+1)H(k+1)]A(k+1,k)\\ \;\;\;\;\times [P(k|k-1)H^{T}(k)-K(k)C_{0}(k)]\\ \forall j=1,2,3,\cdots \end{array}.$$

In general, $C_{j}(k)$ in Equation (25) is equal to zero when Equations (20) and (24) are substituted into Equation (25), implying that the residual sequences are uncorrelated when the optimal gain is applied. However, the actual covariance of the residual $C_{0}(k)$ is different from the theoretical covariance, owing to errors in the system model parameters and noise covariance. Therefore, $C_{j}(k)$ may not be equal to zero. In Equation (25), we can choose a forgetting factor such that the last term of $C_{j}(k)$ for all is zero.

(26) $$P(k|k-1)H^{T}(k)-K(k)C_{0}(k)=0.$$

In the optimal condition, S(k) and g(λ,k) are as follows:

(27) $$S(k)=P(k|k-1)H^{T}(k)-K(k)C_{0}(k),$$

(28) $$g(\lambda ,k)=\frac{1}{2}\sum_{i=1}^{n}\sum_{j=1}^{m}S_{ij}^{2}(k).$$

The optimality of the Kalman filter can be determined through Equation (27), which is a scalar function, and $S_{ij}(k)$ is (i,j) th element of S(k). As the smaller g(k) yields more optimal filter, the forgetting factor λ(k) should be selected to minimize g(k).

Various studies have been conducted based on the least-squares estimation (LSE) approach to better track time-varying parameters of dynamic systems. In this study, a recursive estimation method with a forgetting factor update was introduced to track time-varying parameters. The constant forgetting factor was optimally updated based on the following equation (i.e., the gradient descent method):

(29) $$\lambda^{l+1}(k)=\lambda^{l}(k)+\varphi \frac{\partial g^{l}(\lambda ,k)}{\partial \lambda^{l}(k)}\forall l=0,1,2,\ldots ,$$

with initial conditions

(30) $$\lambda^{0}(1)=1,\lambda^{0}(k)=\lambda (k-1),$$

where *k* is the time series and *l* is the iteration time of the time instant. *φ* is the step length (i.e., learning rate, 0<φ<1). If Equation (31) is satisfied in the *p*-th iteration (i.e., converges), the iteration is stopped, and the optimal forgetting factor is determined using Equation (32).

(31) $$\left|\lambda^{p}(k)-\lambda^{p-1}(k)\right|<\varepsilon .$$

(32) $$\lambda (k)=\max\{1,\lambda^{p}(k)\}.$$

However, the iterative numerical method does not guarantee real-time processing. Finally, a one-step AEFK algorithm was used to resolve this computational burden. In the given system, state Equations (12), (18), and (19) have the following assumption:

**Assumption 1.**  Q(k),R(k) and P(0) *are positive definite.*

**Assumption 2.**  *The measurement matrix*H(k)*is fully ranked, and the optimal forgetting factor can be calculated as*

(33) $$\lambda (k)=\max\left\{1,trace[N(k)]/trace[M(k)]\right\},$$

*where*

(34) $$M(k)=H(k)A(k,k-1)P(k-1)A^{T}(k,k-1)H^{T}(k),$$

(35) $$N(k)=C_{0}(k)-H(k)Q(k-1)H^{T}(k)-R(k).$$

The $C_{0}(k)$ value was estimated using the recursive equation as follows:

(36) $$C_{0}(k)=G_{1}(k)/G_{2}(k),$$

(37) $$G_{1}(k)=G_{1}(k-1)/\lambda (k-1)+z(k)z^{T}(k),$$

(38) $$G_{2}(k)=G_{2}(k-1)/\lambda (k-1)+1$$

with initial conditions $G_{1}(0)=0$ and $G_{2}(0)=0$. The proofs of Equations (33)–(35) was derived by substituting Equation (20), which derives the Kalman gain value into Equation (26).

(39) $$\begin{array}{l} P(k|k-1)H^{T}(k)\\ \times \{I-{\left[H(k)P(k|k-1)H^{T}(k)+R(k)\right]}^{-1}C_{0}(k)\}=0 \end{array}$$

(40) $$H(k)P(k|k-1)H^{T}(k)=C_{0}(k)-R(k).$$

Equation (40) implies that, with Assumptions 1 and 2, the optimality condition described in Equation (26) is equivalent to Equation (24). Substituting Equation (14) into Equation (40), and then reconstructing it yields the following:

(41) $$\begin{array}{l} \lambda (k)H(k)A(k,k-1)P(k-1)A^{T}(k,k-1)H^{T}(k)\\ =C_{0}(k)-H(k)Q(k-1)H^{T}(k)-R(k) \end{array}.$$

**Figure 3:**
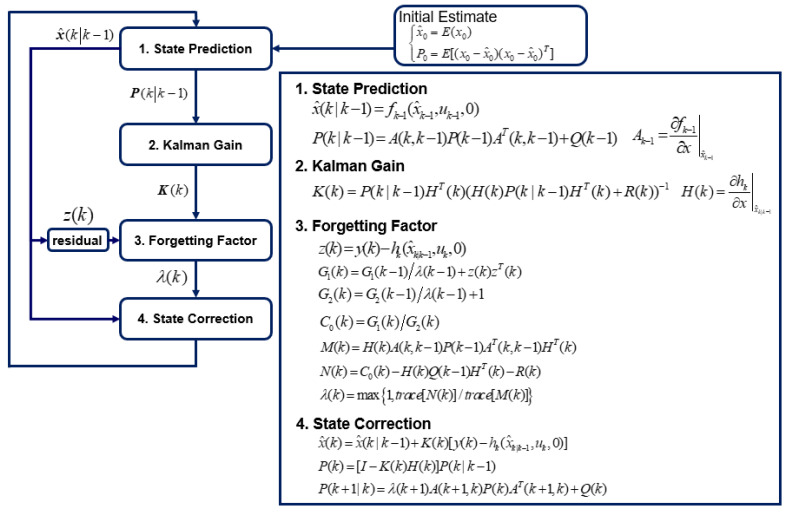
Overall flow chart of the AEKF algorithm for estimating the time-varying shaft torsional stiffness.

The overall estimation process using the AEKF algorithm with a forgetting factor update is shown in Figure 3.

## 3. Estimation of Shaft Torsional Stiffness

### 3.1. Simulation Scheme

Based on the proposed algorithm, a situation in which cracks occur owing to shaft damage was simulated using MATLAB^®^. The parameter values of the system model are listed in Table 1. In this study, to mimic crack fatigue due to persistent cyclic excitation, a sinusoidal torque input was applied (frequency of 1 Hz, $T_{m}(t)=10,000\sin(2\pi t)\;Nmm$). The angular velocity measurement data from the simulation model was set to be contaminated by the white Gaussian random noise $v(k)=N(0,{\left(10^{-3}\right)}^{2})$.

**Figure 4:**
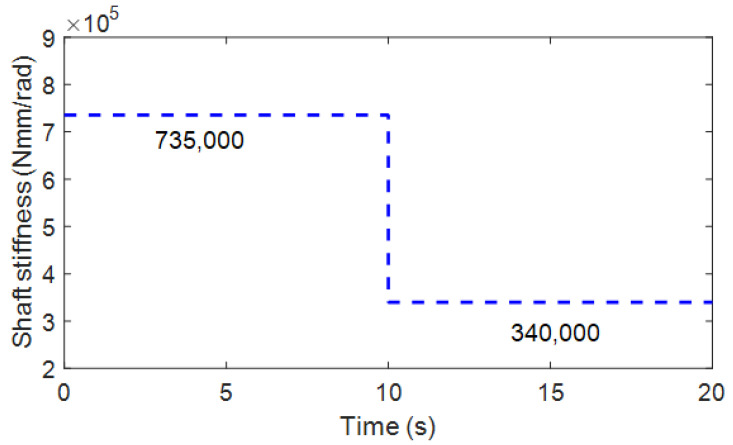
Crack scenario for sudden shaft torsional stiffness drop (735,000 → 340,000 Nmm/rad).

To evaluate the response time of the proposed estimator, step response to a sudden downward step input is used for the crack initiation scenario, which corresponds to large cracks in an experiment. Then, it was assumed that the torsional stiffness suddenly decreased from 735,000 to 345,000 Nmm/rad in 10 s, as shown in Figure 4. 

The initial state value and error covariance for estimation are as follows:

(42) $$\begin{array}{l} x_{0}=\begin{array}{cccc} {}[0 & 0 & 735,000 & 0] \end{array}\\ P_{0}=diag\begin{array}{cccc} ([0.01 & 1 & 800,000 & 1]) \end{array}\; \end{array}.$$

The system noise covariance matrix *Q* and the measurement noise covariance *R* for Equations (21) and (22) were tuned in various cases as follows, and the optimal estimates were derived:

(43) $$Q=\left[\begin{array}{cccc} 10^{-8} & & & \\ & 10^{-7} & & \\ & & 10^{-7} & \\ & & & 10^{-7} \end{array}\right]\;,R=\left[\begin{array}{cc} 10^{-3} & \\ & 10^{-3} \end{array}\right]\;.$$

**Figure 5:**
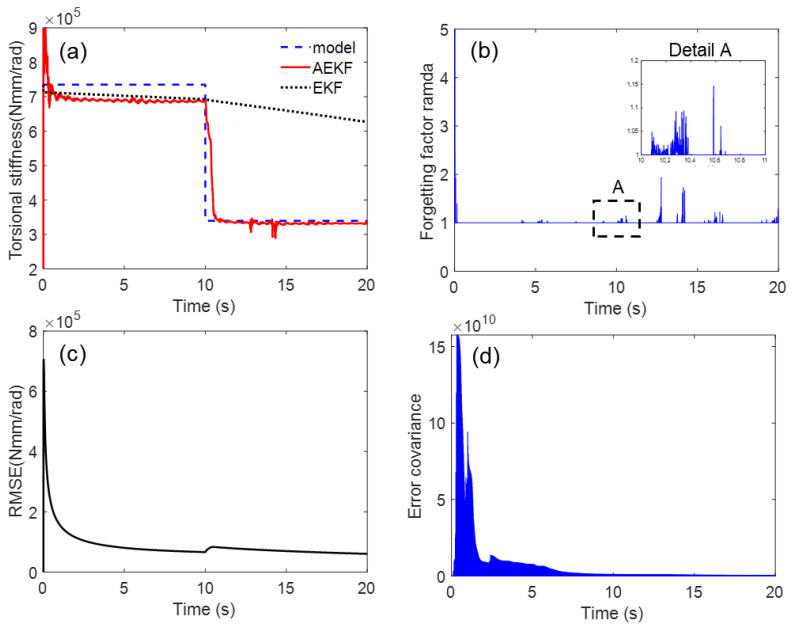
Simulation result for tracking sudden torsional stiffness drop: (**a**) time response of torsional stiffness, (**b**) corresponding time history of forgetting factor, and (**c**) RMSE, (**d**) convergence history of covariance.

**Figure 6:**
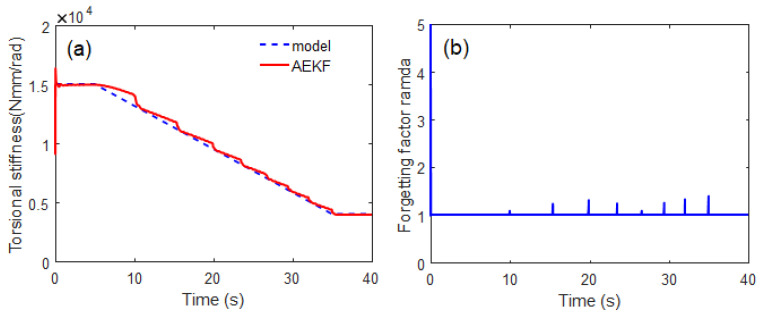
Simulation result for tracking gradual torsional stiffness drop for 30 s: (**a**) time response of torsional stiffness, (**b**) corresponding time history of forgetting factor.

To evaluate the basic estimation performance of the AEKF, the root-mean-squared error (RMSE) at the *k*th time instant was calculated for a more rigorous analysis.

(44) $$RMSE(k)=\sqrt{\frac{1}{k}\sum_{i=1}^{k}{\left(p(i)-\hat{p}(i)\right)}^{2}},$$

where *k* is the time instant at *t = k*Δ*t*, p(i) and $\hat{p}(i)$ are the true (i.e., Figure 4) and estimated values, respectively. The steady-state mean of the RMSE (MRMSE) was then calculated to exclude the effect of transient behavior. The basic estimation results for the sudden torsional stiffness drop are shown in Figure 5. The AEKF accurately estimated the sudden torsional stiffness change. In contrast, the EKF did not track the time-varying shaft stiffness change. The forgetting factor was appropriately changed by the P-adaptive loop when the stiffness rapidly decreased in 10 s. Additional scenarios with different reduction rates are applied to the simulation model to investigate the effectiveness of the proposed algorithm. These different scenarios allow for the evaluation of the tracking performance of the proposed algorithm under the same conditions, such as process and measurement noise covariance matrices. As shown in Figure 6, a gradual reduction from $1.5\times 10^{4}\;\mathrm{Nmm}/\mathrm{rad}$ starts at approximately 5 s, drops to $0.4\times 10^{4}\;\mathrm{Nmm}/\mathrm{rad}$ at 35 s (simulating a situation where the crack is propagating). When a crack growth is propagating and a gradual torsional stiffness drop occurs, the AEKF can deal appropriately.

### 3.2. Robustness Analysis

The robustness of the proposed estimation model under noise and parametric model uncertainty was analyzed by introducing perturbations to sensor noise and main parameters. To evaluate the robustness under noise and parametric uncertainties, the relative error to the nominal value (i.e., normalized performance measure) was quantitatively calculated.

(45) $$\mathrm{Relative}\;\mathrm{Error}=\frac{\left|MRMSE_{\mathrm{Perturbed}}-MRMSE_{\mathrm{Nominal}}\right|}{MRMSE_{\mathrm{Nominal}}}.$$

**Figure 7:**
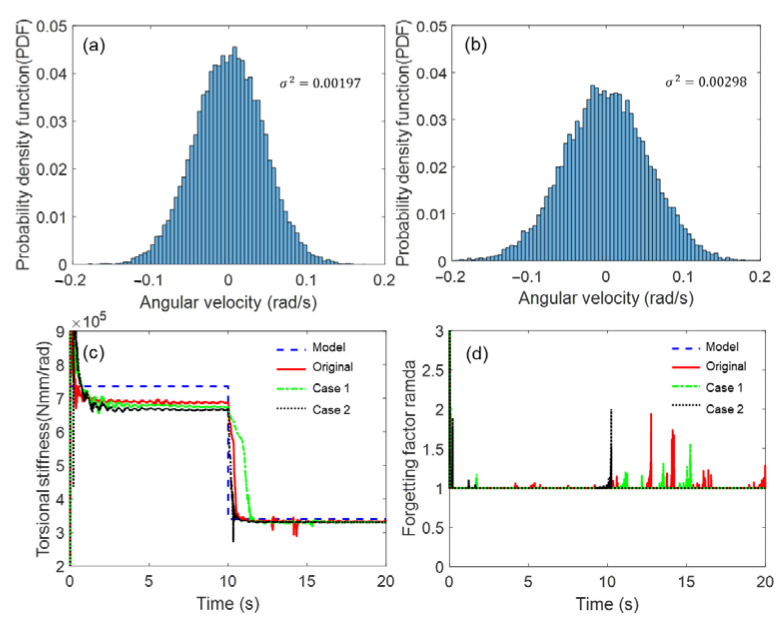
Simulation results of shaft stiffness estimation under noise uncertainty. Gaussian random distribution: (**a**) Case 1, and (**b**) Case 2, (**c**) torsional shaft stiffness, (**d**) forgetting factor.

As sensor information is inherently contaminated by electrical noise, the effect of electrical noise on the estimated performance was examined. The sensor data were contaminated by adding a white Gaussian random noise. A probability density function is shown in Figure 7a,b as an example. Considering the random noise (error) distribution can be fitted to a normal Gaussian distribution with variance ($\sigma^{2}=0.00197$, Case 1; $\sigma^{2}=0.00298$, Case 2), it was confirmed by white Gaussian random noise. The proposed AEKF appeared to be robust against the Gaussian random noise extracted from the sensor data because the estimation results appeared to be similar to the original data, with no significant discrepancy, as shown in Figure 7c,d.

**Figure 8:**
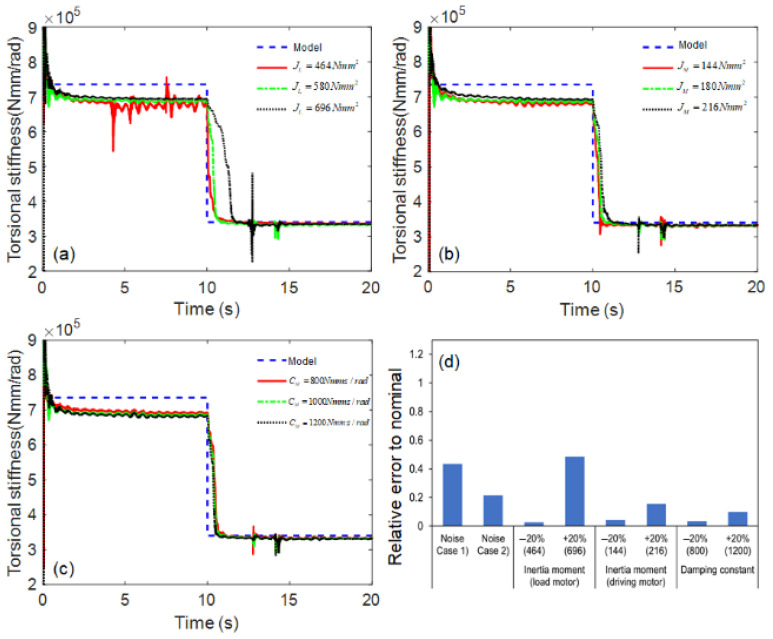
Simulation results of shaft stiffness estimation under parametric uncertainty: (**a**) inertia moment of load motor, (**b**) inertia moment of driving motor, (**c**) damping constant, and (**d**) relative errors.

The estimation performance of the proposed AEKF model was evaluated under parametric uncertainty, such as the moment of inertia. The moment of inertia on both sides is an important model uncertainty because it depends on the size, weight, and connection structure of the coupling. The nominal value for the moment of inertia of the load motor (580 $Nmm^{2}$) was perturbed by −20% (464 $Nmm^{2}$) and +20% (696 $Nmm^{2}$), and the nominal inertia moment of the driving motor (180 $Nmm^{2}$) was also perturbed by −20% (144 $Nmm^{2}$) and +20% (216 $Nmm^{2}$). The damping coefficient varied under normal operating conditions (800~1200 Nmm·s/rad), depending on the bearing lubrication condition. As the estimation results were similar to the nominal values within a reasonable range under various parametric uncertainties, the robustness of the proposed model was demonstrated, as shown in Figure 8.

## 4. Experimental Validation

### 4.1. Experimental Set-Up

**Figure 9:**
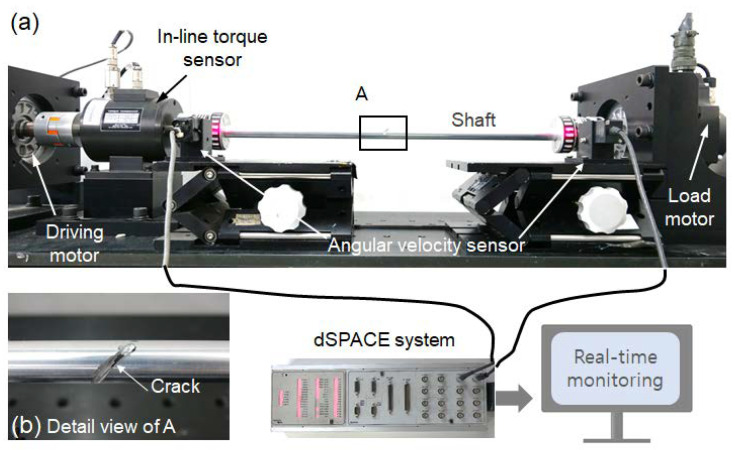
Schematic of experimental set-up: (**a**) overall photograph, and (**b**) details of cracked shaft.

The proposed shaft health-monitoring method was experimentally validated using a torque dynamo. An aluminum hollow-rod specimen ($D_{o}$: 20 mm, $D_{i}$: 18.2 mm, *L*: 550 mm) was used for the rotating shaft, as shown in Figure 9. The torque dynamo comprises a driving motor and torque-controlled load motor (Mitsubishi HG-SR152, 10 Hz bandwidth). Sinusoidal torque ($T_{m}(t)=10,000\sin(2\pi t)$ Nmm) was applied at a rotating speed of 5.23 rad/s (50 RPM). For the shaft crack scenario, the shaft was exchanged in turn from a normal shaft without cracks to a cracked shaft in the 45° direction (Figure 9b). The crack depth was set to 5 mm to ensure that the shaft stiffness could suddenly drop from the original value. To examine the possibility of applying a non-contact angular velocity sensor (tachometer), the measurement model considered the angular velocity values on both sides of the rotating shaft. The angular velocities of both sides were measured using a photoelectric detector-type rotational velocity sensor (ONO SOKKI, model: LG-930), which calculates the rotation speed by counting the light reflected on the gear per rotation as a pulse. The real-time monitoring performance was evaluated using a dSPACE^®^ system (DS1104). 

In this study, the recursive least square estimator (RLSE) was used to identify the unknown model parameters. The rotating shaft model expressed in Equations (1) and (2) were reformulated in the matrix form as follows:

(46) $$y_{k}=h_{k}^{T}\theta_{k}+v_{k}$$

where

(47) $$y_{k}=\left[\begin{array}{c} T_{m}\\ 0 \end{array}\right],\;h_{k}^{T}=\left[\begin{array}{llll} {\ddot{\theta }}_{m} & 0 & {\dot{\theta }}_{m} & \left(\theta_{m}-\theta_{l}\right)\\ 0 & -{\ddot{\theta }}_{l} & 0 & \left(\theta_{m}-\theta_{l}\right) \end{array}\right],\;\theta_{k}={\left[J_{m},\;J_{l},\;c_{m},\;k_{s}\right]}^{T}$$

In addition to the measured data from the sensors, other information was required for the two matrices $y_{k}$ and $h_{k}^{T}$. First, the input torque ($T_{m}$) in matrix $y_{k}$ was measured using an in-line torque sensor (model: YDR-2K), as shown in Figure 9. The angular displacement ($\theta_{m}-\theta_{l}$) and two angular accelerations (${\ddot{\theta }}_{m}$, ${\ddot{\theta }}_{l}$) for the matrix $h_{k}^{T}$ was obtained by directly differentiating and integrating using the low-pass filtering of the angular velocity signal. The RLSE was then designed as follows:

▪ Initial estimates

(48) $${\hat{\theta }}_{0}=E\left[\theta \right]$$

(49) $$P_{0}=E\left[\left(\theta -{\hat{\theta }}_{0}\right){\left(\theta -{\hat{\theta }}_{0}\right)}^{T}\right]$$

▪ Kalman gain calculation

(50) $$K_{k+1}=P_{k}h_{k+1}{\left(h_{k+1}^{T}P_{k}h_{k+1}+w_{k+1}^{-1}\right)}^{-1}$$

▪ Parameter update

(51) $${\hat{\theta }}_{k+1}={\hat{\theta }}_{k}+K_{k+1}\left(y_{k+1}-h_{k+1}^{T}{\hat{\theta }}_{k}\right)$$

▪ Covariance update

(52) $$P_{k+1}=\left(I-K_{k+1}h_{k+1}^{T}\right)P_{k}$$

**Figure 10:**
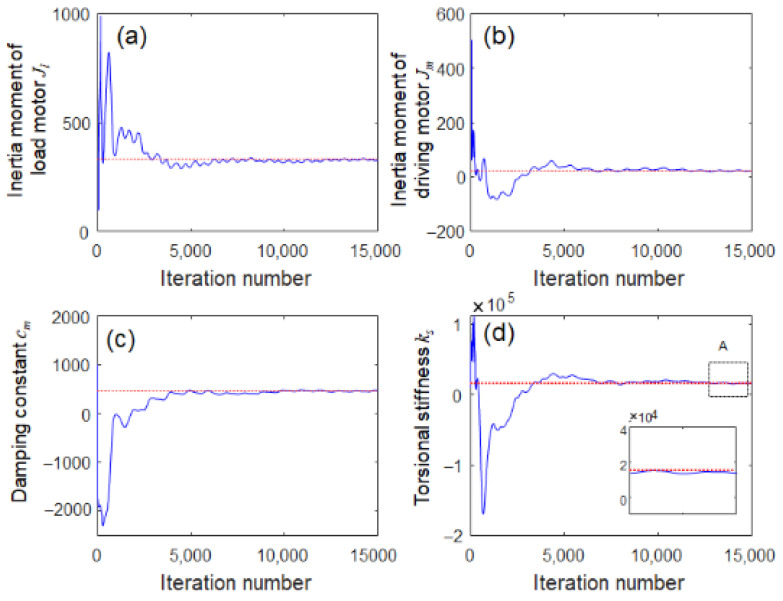
Convergence histories in system identification: (**a**) inertia moment of load motor, (**b**) inertia moment of driving motor, (**c**) damping constant, and (**d**) shaft torsional stiffness (inset: zoomed view of A).

All parameters of the rotating system are successfully estimated because they converge to a steady-state final positive value after 5000 iterations, as shown in Figure 10. The identified system parameters of the rotating shaft model are listed in Table 2.

### 4.2. Results and Discussion

**Figure 11:**
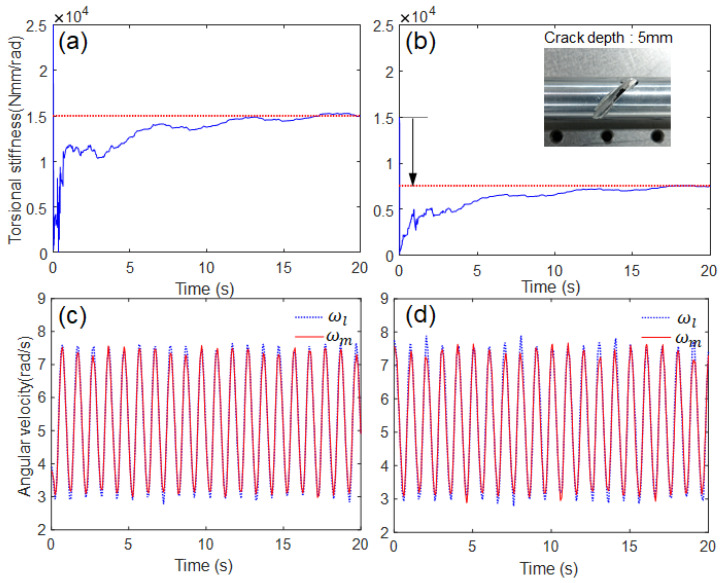
Experimental results: (**a**,**b**) estimated responses of torsional stiffness, (**c**,**d**) angular velocity inputs, (**a**,**c**) without crack, (**b**,**d**) with crack (crack depth 5 mm).

For the AEKF estimation model, the initial states were set, and the two noise covariance matrices (*Q* and R) were tuned by trial and error, as listed in Table 3. The shaft stiffness estimated using the proposed algorithm was compared in Figure 11. The estimated stiffness became steady-state and converged after 15 s in both cases. In the case of the normal state (no crack), the convergence value was identical to the system identification value (i.e., 15,000 Nmm/rad). When the stiffness changes owing to the sudden drop of crack (crack depth 5 mm) from 15,000 Nmm/rad (normal) to a certain value (abnormal crack, in this case approximately 7500), the proposed algorithm can detect this sudden drop. However, it was difficult to conform to the shift in shaft stiffness by naked eyes from the two angular velocity inputs.

**Figure 12:**
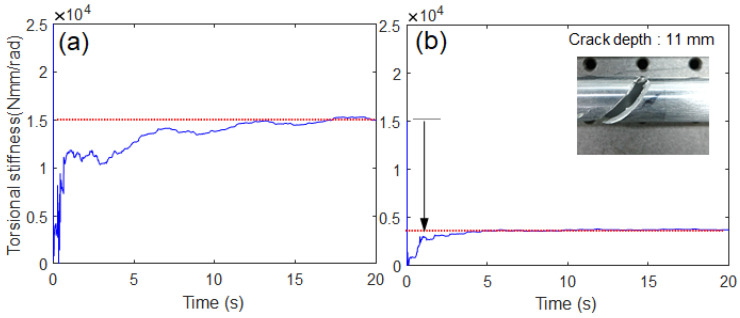
Experimental results for different crack scenario: (**a**) without crack, (**b**) with crack (crack depth: 11 mm) [Supplementary Materials].

**Figure 13:**
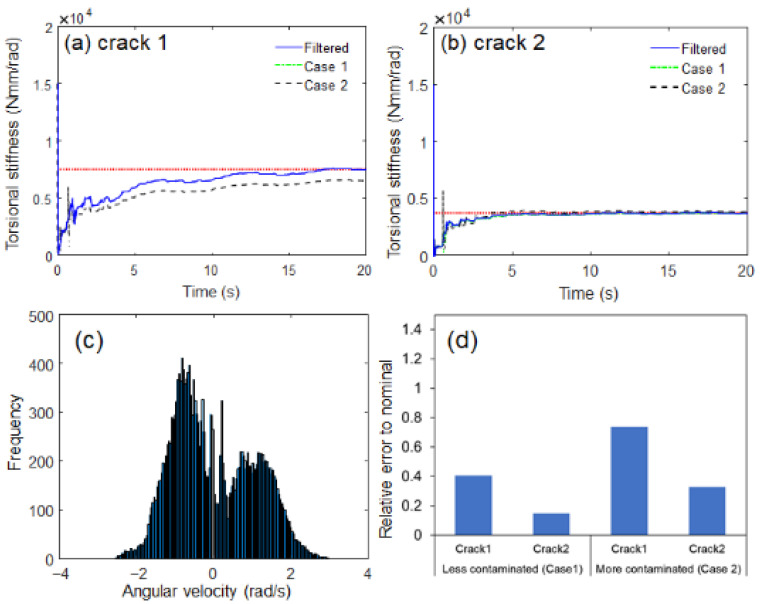
Estimated torsional stiffness responses under electrical sensor noise uncertainty: (**a**) crack depth 5 mm, (**b**) crack depth 11 mm, (**c**) probability density distribution of sensor noise extract from original sensor signal, (**d**) relative error to nominal.

A different crack scenario was established to further investigate the effectiveness of the proposed algorithm. The crack depth was further increased (11 mm) to suddenly drop from 15,000 Nmm/rad to below 7500 Nmm/rad owing to the reduction in cross-sectional area (the ratio of the crack segment area to the original cross-sectional area was 65%) [29,30]. Similar to Figure 11, the proposed algorithm can track this stiffness drop due to the heavy crack, as shown in Figure 12. The proposed estimation model could not only estimate the decrease in stiffness caused by a crack, but also quantitatively evaluate the fatigue crack growth by directly estimating the shaft torsional stiffness. The robustness of the proposed estimation model under noise uncertainty was evaluated by introducing the perturbations in sensor noise. The original sensor signal was filtered by a digital moving average filter (no phase delay). Two corrupted signals were generated; the low-pass filtering is small (Case 1, less contaminated) and off (Case 2, more contaminated, i.e., raw data). The probability density distribution of sensor noise extracted from the original sensor signal was similar to Gaussian distribution, as shown in Figure 13c. The proposed estimation model seemed to be robust against the Gaussian random noise in all sensor data because the estimation results appeared to be similar regardless of the degree of contamination, as shown in Figure 13a,b. In the case of heavy crack (crack depth 11 mm), the proposed estimation model turned out to be more robust against the Gaussian random noise, as shown in Figure 13d. The robustness of the proposed estimation model under model uncertainty was not investigated in the experiment because it was unlikely to significantly change two main model parameters (inertia moment of load and driving motor). 

## 5. Conclusions

In this study, the torsional crack in the rotating shaft was successfully detected in real-time by estimating the reduction of torsional stiffness in the rotating shaft using the AEKF approach with forgetting factor update. The main contributions of this study are summarized as follows:

▪ We concluded that the proposed approach is a promising alternative means for detecting torsional cracks in rotating shafts despite the difficulty in tuning the Q and R matrices of the AEKF.
▪ The proposed estimation model could not only estimate the decrease in stiffness caused by a crack but also quantitatively evaluate the fatigue crack growth by directly estimating the shaft torsional stiffness.
▪ Another advantage of the proposed approach is that it uses only two cost-effective rotational speed sensors; therefore, it does not require noncontact-type torque sensors, which are typically expensive and suffer from durability limitations.

With these advantages, the proposed approach can be readily implemented in structural health monitoring systems of rotating machinery. In future research, we will continue to address some of the ongoing issues. In particular, the localization of cracks in rotating shafts should be studied further. In addition, if the input variables cannot be measured, an advanced algorithm should be applied to simultaneously estimate unknown input and state variables.

## Figures and Tables

**Table 1:** Parameters for the estimation of torsional stiffness.

Parameters (Unit)	Value
Inertia moment of load motor $J_{l}\;(Nmm^{2})$	580
Inertia moment of driving motor $J_{m}\;(Nmm^{2})$	180
Damping constant *c_m_* (Nmm·s/rad)	1000
Shaft torsional stiffness *k_s_* (Nmm/rad)	735,000

**Table 2:** Identified system parameters of the rotating shaft model.

Parameters (Unit)	Value
Inertia moment of load motor $J_{l}({\mathrm{Nmm}}^{2})$	595
Inertia moment of driving motor $J_{m}({\mathrm{Nmm}}^{2})$	20
Damping constant *c_m_* (Nmm·s/rad)	280
Shaft torsional stiffness *k_s_* (Nmm/rad)	15,000

**Table 3:** Tuning parameters for the AEKF estimation model.

*P*_0_	*diag*[0.1 1 650,000 1]
*Q*	*diag*[1 2.1 2 2.1] × 10^−5^
*R*	*diag*[9 9] × 10^−8^

## Data Availability

The datasets used and/or analyzed during the current study are available from the corresponding author upon reasonable request.

## Supplementary Materials

The following are available online at https://www.mdpi.com/article/10.3390/s23052437/s1, Video S1: Crack Monitoring in Rotating Shaft Using Torsional Stiffness Estimation with Adaptive Extended Kalman Filters.
